# Enhancing Antifungal Drug Discovery Through Co-Culture with Antarctic *Streptomyces albidoflavus* Strain CBMAI 1855

**DOI:** 10.3390/ijms252312744

**Published:** 2024-11-27

**Authors:** Ana Luisa Perini Leme Giordano, Marili Villa Nova Rodrigues, Karen Gabriela Araujo dos Santos, Barbara Cipulo Legabão, Lais Pontes, Derlene Attili de Angelis, Fabiana Fantinatti Garboggini, Angelica Zaninelli Schreiber

**Affiliations:** 1 Faculdade de Ciências Médicas, Universidade Estadual de Campinas, Campinas 13083-970, SP, Brazil; analuisa.giordano@gmail.com (A.L.P.L.G.); karen.gabrielasantos@outlook.com (K.G.A.d.S.); bcipulo02@gmail.com (B.C.L.); ponteslaais@gmail.com (L.P.); 2 Centro Pluridisciplinar de Pesquisas Químicas, Biológicas e Agrícolas (CPQBA), Universidade Estadual de Campinas, Paulínia 13083-970, SP, Brazil; marilivn@unicamp.br (M.V.N.R.); derlene@cpqba.unicamp.br (D.A.d.A.); fabianaf@unicamp.br (F.F.G.)

**Keywords:** natural products, metabolomics, genome mining, antifungal drugs, fungal infections

## Abstract

Fungal infections pose a growing public health threat, creating an urgent clinical need for new antifungals. Natural products (NPs) from organisms in extreme environments are a promising source for novel drugs. *Streptomyces albidoflavus* CBMAI 1855 exhibited significant potential in this regard. This study aimed to (1) assess the antifungal spectrum of the CBMAI 1855 extract against key human pathogens, (2) elicit NP production through co-cultivation with fungi, correlating the metabolites with the biosynthetic gene clusters (BGCs), and (3) perform in silico toxicity predictions of the identified compounds to analyze their suitability for drug development. The crude extract of CBMAI 1855 exhibited broad-spectrum antifungal activity. The metabolomic analysis identified antifungal NPs such as antimycin A, fungimycin, surugamides, 9-(4-aminophenyl)-3,7-dihydroxy-2,4,6-trimethyl-9-oxo-nonoic acid, and ikarugamycin, with the latter two predicted to be the most suitable for drug development. Genome mining revealed three cryptic BGCs potentially encoding novel antifungals. These BGCs warrant a detailed investigation to elucidate their metabolic products and harness their potential. CBMAI 1855 is a prolific producer of multiple antifungal agents, offering a valuable source for drug discovery. This study highlights the importance of exploring microbial interactions to uncover therapeutics against fungal infections, with a detailed exploration of cryptic BGCs offering a pathway to novel antifungal compounds.

## 1. Introduction

Fungal infections are recognized as neglected diseases, disproportionately affecting immunocompromised individuals, such as those with HIV, cancer, organ transplants, and chronic illnesses like diabetes. The annual incidence of invasive fungal infections (IFIs) is estimated at 6.5 million cases, resulting in approximately 3.8 million deaths [1,2,3,4]. The incidence of susceptible people is rising, driven by medical advancements (e.g., development of oncological treatments, immunosuppressive therapies, and invasive surgical procedures) and climate change, facilitating the geographic spread and prevalence of fungal pathogens [2,3,4,5]. Major fungal diseases include candidiasis, aspergillosis, and cryptococcosis. Additionally, the emergence of *Candida auris*, a multidrug-resistant yeast, is linked to rising global temperatures and represents a growing threat [3,6,7]. Antifungal resistance is a significant concern that complicates treatment and highlights the need for new antifungal agents [8,9].

Despite their increasing impact, fungal diseases remain underreported, underdiagnosed, and under-researched, amplifying their burden. The antifungal pharmacopeia is limited, starkly contrasting with the more extensive arsenal available for bacterial infections [10]. For IFIs, there are four major classes of antifungal agents: polyenes, azoles, echinocandins, and the pyrimidine analog 5-flucytosine (5-FC), with a fifth class, the triterpenoid ibrexafungerp, in advanced clinical trials [11,12]. Polyenes, like amphotericin B (AMB), developed in the 1950s, are potent and broad-spectrum but have significant host toxicities and poor oral bioavailability [13]. The pyrimidine analog 5-FC, approved in the 1960s, has limited use as a monotherapy [14]. Azoles, introduced in the 1980s, inhibit ergosterol biosynthesis and have favorable safety and bioavailability profiles, though they can induce hepatotoxicity; their emerging resistance is also concerning [15,16]. Echinocandins, developed in the early 2000s, inhibit 1,3-β-D-glucan synthase, crucial for fungal cell wall synthesis, and have a favorable safety profile, making them an adequate first-line treatment for invasive candidiasis; however, they are ineffective against other key pathogens [17,18].

Aside from the toxicity, limited administration routes, and the shortage of available antifungal classes, there are additional complexities to be considered. The spectrum of activity of antifungals is crucial in determining how well they combat infections [19]. Many antifungals have a narrow spectrum of activity, which poses significant challenges in clinical settings. The drugs available primarily target specific fungal groups or species [20,21]. For example, FCZ effectively treats candidiasis but may not work against other fungi like molds, *Cryptococcus* presents innate resistance to echinocandins, and *C. auris* can be multidrug-resistant. Furthermore, the intrinsic resistance of certain fungi adds complexity to treatment strategies [22,23]. This resistance, stemming from natural fungal traits such as cell wall composition or efflux pump mechanisms, can render antifungals ineffective against certain species [22]. Consequently, treatment options are limited, contributing to treatment failures. Addressing these challenges requires the discovery of new fungicides with a broad spectrum of activity.

Antifungal drug development is markedly slower compared to other therapeutic fields, representing one critical challenge in managing fungal infections. This delay is fundamentally rooted in the evolutionary proximity between fungal pathogens and their human hosts, which are both eukaryotic organisms [24,25]. This relationship results in the conservation of many biochemical and cellular processes, thereby increasing the likelihood that compounds toxic to fungi will also be toxic to human cells. The existing antifungal drug classes target fungal structures such as ergosterol in the cell membrane and β-glucan in the cell wall, which are absent in human cells. Despite this specificity, these drugs can still induce adverse effects in humans due to off-target interactions or toxicity [12,24,25]. A comprehensive antifungal armamentarium is indispensable for selecting the optimal therapeutic regimen for individual patients. Considering the escalating fungal resistance, there is a pressing need to develop additional therapeutic options. More research is needed to uncover new antifungal agents [11,24].

In the realm of antifungal drug discovery, microbial natural products (NPs) are up-and-coming due to their demonstrated efficacy and structural diversity [26,27,28]. Several pivotal antifungal agents, such as the polyenes AMB, nystatin, and candicidin, are derived from microbial sources, specifically from the genus *Streptomyces*. AMB is a cornerstone in treating severe systemic fungal infections, nystatin is essential for addressing cutaneous and mucosal candidiasis, and candicidin is also used in the topical treatment of vulvovaginal candidiasis [29,30,31]. Notably, microorganisms from extreme environments, such as Antarctica, are promising candidates for novel drug discovery. The unique and often harsh conditions of these environments drive the evolution of distinctive metabolic pathways and biochemical adaptations, increasing the likelihood of uncovering unprecedented molecular structures with varied bioactivity [28,31,32]. This untapped reservoir of microbial diversity holds the potential for developing innovative therapeutics, addressing the unmet clinical need for new treatments in the face of rising antifungal resistance [10,32].

*Streptomyces albidoflavus* CBMAI 1855, isolated from the tunicate *Salpa* sp. in King George Island, Antarctica, has been previously investigated for its diverse bioactivities, as demonstrated by França et al. (2023) [33]. Their study highlighted the bacterium’s potential through organic crude extracts that exhibited antibacterial activity against various pathogens and antiproliferative effects against multiple cancer cell lines. These findings uncovered CBMAI 1855’s rich biosynthetic potential and its relevance in biotechnological applications. Despite these advancements, the exploration of CBMAI 1855′s antifungal activity remains underexplored. Considering its bioactivity against *Candida albicans* ATCC 10231, investigating the breadth and efficacy of CBMAI 1855′s antifungal properties is crucial [33]. Given the critical public health implications of fungal infections and the potential of this strain, it is imperative to assess its antifungal activity spectrum comprehensively. Investigating whether its antifungal properties extend beyond *C. albicans* to other public health-relevant fungal species is essential for harnessing the full therapeutic potential of CBMAI 1855 as a source of broad-spectrum antifungal agents.

A previous metabologenomic analysis demonstrated the high biosynthetic potential of CBMAI 1855. The documented bioactivity against *Candida albicans* ATCC 10231 highlights the strain’s potential to produce antifungal compounds [33]. However, the metabolomic analysis under monoculture conditions did not fully explain which metabolites are responsible for the observed antifungal activity. Additionally, the fact that CBMAI 1855 was isolated from the extreme environment of Antarctica increases the likelihood of producing novel molecules with unique bioactivities.

In this study, we aim to build upon previous research by investigating the Antarctic bacterium *Streptomyces albidoflavus* CBMAI 1855′s antifungal capabilities in greater detail. Our objectives are threefold: (1) to assess the spectrum of its antifungal activity against a comprehensive panel of fungal pathogens of public health importance, (2) to explore strategies for eliciting the production of antifungal metabolites through the co-cultivation of CBMAI 1855 with fungi, and (3) to conduct in silico toxicity predictions of the identified compounds. By elucidating the antifungal profile of CBMAI 1855, optimizing culture conditions to maximize metabolite yield, and activating biosynthetic gene clusters (BGCs) that encode antifungal metabolites and may be silenced when the bacterium is cultured alone, we aimed to discover novel antifungal agents with therapeutic potential.

## 2. Results

### 2.1. Bioactivity Assays

**Figure 1:**
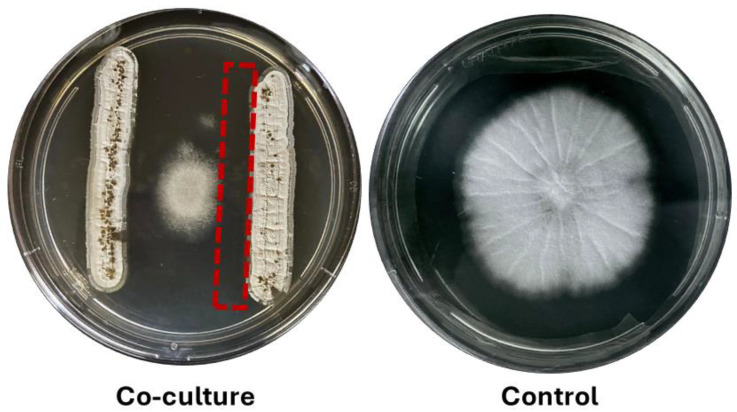
Co-culture of CBMAI 1855 and *Aspergillus flavus* ATCC 204304 after 5 days of fungal inoculation. The inhibition zone, indicating antifungal activity, is highlighted by the red dashed lines.

CBMAI 1855 exhibited robust activity against all tested fungal isolates. In the antagonism test, clear inhibition zones surrounding CBMAI 1855 streaks on Mueller Hinton Agar (MHA) plates indicate inhibitory effects against fungi. These zones were observed during co-culture assays, where CBMAI 1855 was cultured alongside *Aspergillus flavus* ATCC 204304. The inhibition zones, marked in red in Figure 1, delineate regions where the growth of *A. flavus* was inhibited. This inhibition is attributed to the diffusion of antifungal metabolites produced by CBMAI 1855 through the agar medium. These specific regions, where fungal growth was impeded, were collected for metabolomic analysis to identify the bacterial metabolites responsible for this activity. The results of this analysis are presented in Section 2.2.

The broth microdilution assays revealed consistent minimum inhibitory concentration (MIC) values of 1.5 mg/mL for all fungal isolates, including standard ATCC strains as well as both susceptible and resistant clinical isolates (Table 1). However, the Minimum Fungicidal Concentration (MFC) values exhibited deviation for *Cryptococcus* species, indicating that while 1.5 mg/mL effectively inhibited growth, it was insufficient to achieve fungicidal effects for these strains. The results of the broth microdilution assays of the ethyl acetate extract, along with the MIC values of all isolates against commercial antifungal drugs, are summarized in Table 1.

The bioactivity assays demonstrated that the CBMAI 1855 crude extract is active against a broad spectrum of fungal pathogens, as evidenced by the antagonism test result and consistent MIC values across diverse isolates, including the previously accessed MIC of 1.5 mg/mL against *C. albicans* ATCC 10231 [33]. The observed deviations in MEC values for *Cryptococcus* species highlight the challenges posed by the fungal capsule [34,35], which can limit the penetration and efficacy of bioactive molecules present in the extract.

In comparison with commercial antifungal drugs used as positive controls, CBMAI 1855 extracts demonstrated effects irrespective of the resistance profiles or susceptibility characteristics of the tested microorganisms. This consistency reinforces the broad-spectrum activity of CBMAI 1855-derived compounds, which likely arises from the presence of multiple antifungal molecules within the extract, each with distinct mechanisms of action. Since the extract represents a mixture of all the molecules produced by the bacterium, future studies must isolate individual compounds to determine their MICs and specific mechanisms of action. The higher MFC values observed for *Cryptococcus* species suggest that the fungal capsule may protect these pathogens from the action of CBMAI 1855-derived metabolites.

### 2.2. Metabolomic Analysis

The co-culture approach employed in this study enabled the putative annotation of several antifungal metabolites produced by CBMAI 1855, demonstrating the utility of combining microbial interactions with advanced analytical techniques. By leveraging the GNPS and additional tools within the platform, such as molecular networking (MN) feature-based molecular networking (FBMN), and DEREPLICATOR plus, we were able to analyze and visualize the chemical diversity present in our samples. This methodology has also facilitated the detection of structural analogs. The known antifungal compounds annotated are listed in Table 2. The corresponding mirror match results for these metabolite annotations are provided in Figures S2–S7. Additionally, a segment of the MN related to these metabolites is depicted in Figure 1, with the complete MN data available in Figure S1.

**Figure 2:**
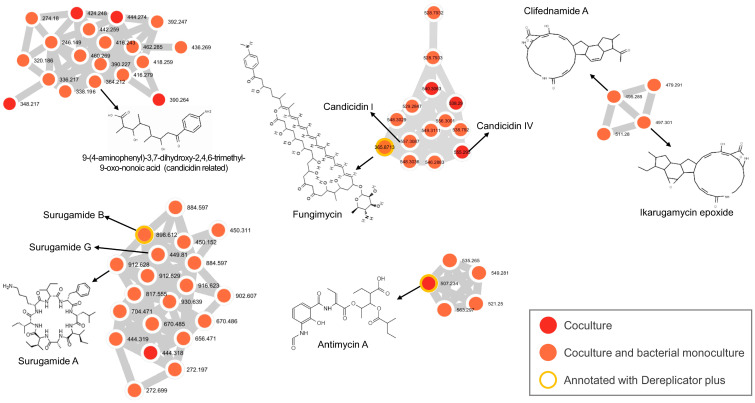
A segment of the molecular network highlighting nodes corresponding to putatively annotated antifungal natural products. The network was constructed from the analysis of sample extracts, with solvent blanks removed. Each node represents the consensus spectra of an ion mass that was detected and is color-coded based on the sample in which it was detected.

Using the GNPS and its features tools, we annotated the compound “9-(4-aminophenyl)-3,7-dihydroxy-2,4,6-trimethyl-9-oxo-nonoic acid” (compound **1**), referred to as a candicidin-related compound. It was detected with a cosine score of 0.88, indicating a high degree of spectral similarity to known compounds within the GNPS database. This compound falls within the polyketide’s natural product pathway, specifically classified under linear polyketides and open-chain polyketides. The detection of compound **1** was reported by Wang et al. (2010) [36]. This compound was produced from cultures of an endophyte, *Streptomyces* sp., obtained from *Aegiceras corniculatum* (Ericales), a mangrove plant. The authors isolated the compound as a yellow oil and found that it exhibited no cytotoxicity against HeLa cell lines [36]. Additionally, the compound was annotated by Moreira et al. (2022) from a *Streptomyces* sp. culture, isolated from the northeastern coast of Brazil [37]. Also, through FBMN, two additional compounds, candicidin I and candicidin IV (compound **2** and **3**, respectively), were identified in the Analog Library Hits with cosine scores of 0.74 and 0.71, respectively. These compounds were recognized as structural analogs based on their MS/MS spectra. However, it is noteworthy that compound **1** did not cluster with compounds **2** and **3** (Figure 2). This separation in clustering suggests distinct structural differences or fragmentation patterns, despite their related annotation.

Interestingly, fungimycin (compound **8**), identified using DEREPLICATOR+, was found to be in the same cluster as compounds **2** and **3**. Fungimycin is a polyene macrolide, like candicidin, and shares the characteristic structural framework of this class [38]. Compound **8** possesses a complex molecular formula of C_59_H_86_N_2_O_17_. Although it is a known polyene macrolide, it has not been considered in drug therapy for mycoses due to its high toxicity (mainly nephrotoxicity) and poor acceptability when systemically administered. This high toxicity differentiates fungimycin from other polyenes such as AMB and candicidin, which are more suitable for medical use [39].

The detection of these three distinct candicidin-related molecules and compound **8** highlights the potential for CBMAI 1855 to produce structurally diverse polyenes within the candicidin family, suggesting that it can represent a novel and previously unknown analog. Given the established antifungal properties associated with candicidin compounds, these findings represent the prospect of exploring new biological activities associated with potentially novel molecules. In the molecular networking (Figure 2), we observe among the compounds **1**, **2**, **3**, and **8**, nodes representing compounds produced exclusively by CBMAI 1855 under co-culture conditions that do not have any annotations, indicating they are unknown. These findings further highlight the potential for discovering novel biological activities associated with these still-unknown polyene molecules. To fully elucidate the structure and confirm the functional properties of the detected molecules, further analyses are necessary. Despite its unsuitability for medical purposes, compound **8** can be considered a valuable antifungal preparation for non-medical uses, such as in agriculture [39]. One of the most important requirements for antifungals in agriculture and the food industry is that they should not be used in medical practice, to avoid resistance (similar to the case with azoles, which are widely used in both medical and agricultural settings) [40,41]. These unknown polyenes macrolides produced by CBMAI 1855 present intriguing prospects, as they may possess more favorable properties for medical applications or effective antifungal action in agricultural contexts. The discovery of such novel molecules could lead to the development of new antifungal agents with potentially lower toxicity and broader applicability for medical, animal, and non-medical purposes.

Like compound **8**, antimycin A (compound **4**) was annotated through the complementary tool DEREPLICATOR+ within the GNPS platform, which employs predicted molecular features and spectral data to aid in compound annotation [42]. Notably, compound **4** was exclusively detected in the co-culture sample of CBMAI 1855 and not in its axenic culture, highlighting its production in response to the interaction with the fungus. This compound is renowned for its anticancer and antimicrobial activities, including potent antifungal action by inhibiting the electron respiratory chain in mitochondria [43,44].

Surugamide A (compound **5**), confidently annotated in GNPS with a high cosine score of 0.91, belongs to the cyclic peptide class within the surugamide family. The related compounds surugamide B and G were also annotated (Figure 2). Compound **5** and surugamide G were previously identified as products of CBMAI 1855 in earlier studies [33], while surugamide G was additionally identified in this study through DEREPLICATOR+. While surugamide A is noted in the literature for its documented antifungal properties, surugamide B and G are not reported to exhibit antifungal activity, suggesting potential differences in their biological roles and activities within microbial communities [45].

Ikarugamycin epoxide and clifednamide A (compounds **7** and **6**) are polycyclic tetramate macrolactams (PTMs), putatively annotated with moderate cosine scores of 0.76 and 0.79 in GNPS, respectively. Compound **6** is a derivative of compound **7** [46]. Both ikarugamycin and its derivatives are reported to possess antifungal, antibacterial, and anticancer activities. Clifednamide A has been shown to have activity against carcinoma cells [46,47,48]. The putative annotation of these molecules can also elucidate the multiple bioactivities observed in CBMAI 1855 [33], contributing to its antifungal, antibacterial, and anticancer potential.

Co-culture was a strategic choice for drug discovery due to its ability to simulate natural microbial interactions, thereby inducing the production of diverse secondary metabolites that are often not expressed in axenic cultures [49]. For instance, compounds **3** and **4** were only detected in the co-culture treatment, highlighting the effectiveness of this method in revealing bioactive compounds. Additionally, several unknown ions were exclusively found in co-culture samples, including those within clusters of known molecules, such as compounds **1** and **8** clusters, where numerous ions lacked annotations in GNPS. These unidentified ions could serve as potential drugs or possess medically advantageous characteristics, demonstrating the promising potential of co-culture in drug discovery.

**Figure 3:**
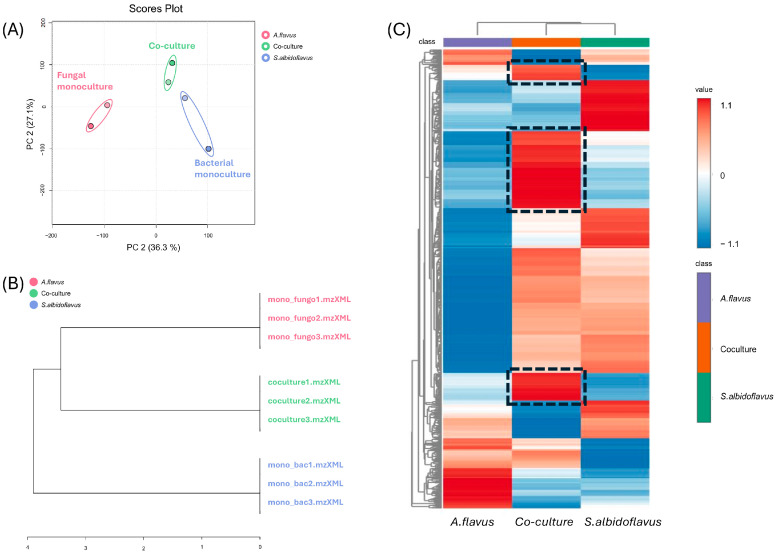
PCA scores plot (**A**); Hierarchical Clustering Dendrogram (**B**), grouping the triplicates of each treatment; and heatmap generated from the HCA of the most significant ions (*p* < 0.05) present in the samples (**C**).

We conducted a statistical analysis of the data to compare the metabolomic profiles of the three conditions and the compounds present in each. The results of the Principal Component Analysis (PCA) and Hierarchical Cluster Analysis (HCA) based on the quantification of ions in our samples are presented in Figure 3. The PCA accounted for 63.4% of the total variance observed among the samples, with PC1 explaining 36.3% and PC2 explaining 27.1% of the variance. As shown in the PCA’s scores plot (Figure 3A) and corroborated by the hierarchical clustering dendrogram (Figure 3B), the PCA effectively reduced the dimensionality of the data, combining the triplicates of each condition into two data points. The Hierarchical Cluster Dendrogram (Figure 3B) and PCA plots (Figure 3A) reveal distinct groupings corresponding to each experimental condition: co-culture (green), bacterial monoculture (blue), and fungal monoculture (pink). The clustering within each treatment condition indicates the high reproducibility of the experimental setup. The PCA’s reduction of dimensionality to two principal components further confirms the reproducibility of the data.

HCA (Figure 3C) was performed on the most significant ions (*p* < 0.05) to construct a heatmap, illustrating ions of high abundance in warm colors and ions of low abundance in cold colors. The analysis revealed that some ions produced in co-culture were markedly distinct from those in bacterial and fungal monocultures. Three clusters of ions were demonstrated with higher abundance in the co-culture condition compared to the monocultures. This differentiation suggests that co-culture environments influence the production of metabolites. The co-culture samples displayed intergroup differences when compared to both bacterial and fungal monocultures, reinforcing that co-cultivation can stimulate the production of a broader array of metabolites.

These findings illustrate the utility of co-culture, molecular networking, and its complementary tools like DEREPLICATOR+ and FBMN in uncovering diverse antifungal NPs. The metabolomic analysis of the co-culture, rather than the axenic culture, enabled us to identify CBMAI 1855 as a multiple antifungal producer. Our results emphasize the potential of co-culture techniques to enhance chemical diversity and elicit the production of specialized metabolites.

### 2.3. Antifungal Genome Mining

**Figure 4:**
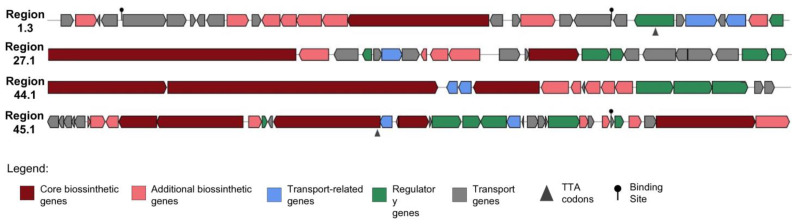
Schematic diagram of the four BGCs that were expressed and showed the highest similarity with the MiBIG database. antiSMASH was used to predict potential secondary metabolite BGCs. Different color blocks represent genes with different functions.

Corroborating with the findings from the metabolomic analysis, the antifungal genome mining also indicates that CBMAI 1855 has significant potential to encode various antifungal compounds. The antiSMASH analysis of the CBMAI 1855 genome (Table S1) reveals several regions with significant biosynthetic potential for producing these compounds. Through the co-culture approach, we were able to express almost all antifungal BGCs (marked in bold in Table 3 and the most similar presented in Figure 4) and correlate the metabolites produced. Table 3 lists ten regions, each associated with different BGCs and their closest known analogs from the MiBIG database.

Region 1.1, which is an NRPS cluster, shows a 66% similarity to the diisonitrile antibiotic SF2768 BGC. SF2768 was originally isolated from *Streptomyces* sp., known for its antibacterial and antifungal properties [50]. This moderate similarity indicates that the BGC shares some significant features with the diisonitrile antibiotic SF2768 cluster, but there are also considerable differences. These differences might lead to the production of a related but distinct compound with potentially unique properties. Therefore, the unknown compounds produced could have similar antifungal activity but possibly with different efficacy or a novel mechanism of action. The moderate similarity allows some functional predictions based on the known cluster, but experimental validation would be necessary to confirm the exact nature and the produced metabolites. SF2369, a SF2768-related compound, had also been identified earlier as an antifungal from *Actinomadura* sp. [51]. Despite not annotating these compounds or known related analogs in our metabolomic analysis, investigating this BGC could lead to significant antimicrobial drug discovery. This is particularly promising given that the source of this compound is a microorganism from an extreme environment, which may contribute to the discovery of unknown molecules with unique bioactivities.

In our metabolomic analysis, we putatively annotated an antifungal metabolite and its analog: compounds **7** and **6**, respectively, which are both PTMs. The cluster that likely encodes these metabolites is found in region 1.3 (Table 3 and Figure 4). This region is characterized as a T1PKS-NRPS hybrid cluster, spanning from 423,354 to 472,764 base pairs, and shows 100% similarity to known clusters associated with SGR PTMs, indicating that it is highly likely to produce similar PTMs compounds. The presence of this cluster and its 100% similarity to known PTM clusters suggest that it is the genetic basis for producing compounds **6** and **7** in our culture conditions. Given the complete match with known PTM clusters, this region’s expression aligns well with the detection of the related metabolites in our analysis. This highlights the cluster’s capability to produce structurally related compounds, further confirming the genetic potential of CBMAI 1855 to synthesize these bioactive PTMs. The high similarity also suggests that these molecules might exhibit similar biological activities, such as antifungal, antibacterial, or anticancer properties, which are typical of PTMs [47].

antiSMASH revealed three distinct regions, namely region 24.1, region 25.1, and region 27.1, which show varying degrees of similarity to known surugamide A/surugamide D BGCs. Region 24.1 exhibited a 42% similarity, region 25.1 showed a 19% similarity, and region 27.1 demonstrated a 61% similarity to these clusters. These findings are reinforced by the detection of compound **5**, surugamide B, and surugamide G in our metabolomic analysis, indicating CBMAI 1855′s capability to produce structurally diverse surugamide analogs with potential novel biological activities and biotechnological applications. Moreover, while surugamide production is commonly associated with marine-derived *Streptomyces* isolates, reports indicate its presence in several *S. albidoflavus* and closely related strains. This suggests that surugamide BGCs may be prevalent among *albidoflavus* species isolates. Almeida et al. (2019) proposed this hypothesis based on their identification of similar BGCs in genomes across the *albidoflavus* phylogroup [52].

Region 40.1 of CBMAI 1855 was identified as harboring a Type III polyketide synthase cluster with 100% similarity to the naringenin biosynthetic gene cluster. This genetic finding suggests that CBMAI 1855 possesses the capability to produce naringenin, a flavonoid compound documented in the literature for its antifungal properties [53]. However, despite this genetic capacity, naringenin was not detected in our metabolomic analysis of CBMAI 1855. Several factors could contribute to this. The conditions used for metabolite extraction and analysis may not be optimal for capturing naringenin, which might have specific biosynthesis and accumulation requirements not met under the experimental conditions. Alternatively, naringenin production by CBMAI 1855 might be regulated or induced under specific environmental stimuli or growth phases not replicated in our study. This highlights the complex interplay between genetic potential and environmental factors in determining metabolite production in microbial systems. Further investigation into the regulatory mechanisms and environmental cues influencing naringenin biosynthesis in CBMAI 1855 is warranted to elucidate its biosynthetic capabilities and potential biotechnological applications fully.

Three contiguous regions of interest were identified, each potentially involved in the biosynthesis of polyene macrolides. Region 40.2 and region 44.1 are both Type I polyketide synthase clusters, with region 40.2 showing a 33% similarity and region 44.1 displaying a high similarity (90%) to the candicidin BGC. Also, region 41.1, identified as a T1PKS cluster, exhibits a 26% similarity to the rosamicin BGC, indicating potential involvement in the biosynthesis of macrolide compounds akin to rosamicin, though with considerable structural divergence. These clusters are closely situated, suggesting possible coordination or interaction in the biosynthesis pathway of polyene macrolide compounds. Despite the lower similarity scores, regions 40.2 and 41.1 are of particular interest. Clusters with low similarity often encode enzymes that lead to significant structural variations in the resulting metabolites, potentially resulting in new and enhanced biological activities. The proximity of these clusters and the putative annotation of other polyene macrolides (compound **8**) and candicidin suggest that these regions may be promising targets for discovering novel antifungal drugs.

The antiSMASH analysis showed a complex BGC in region 45.1, encompassing T1PKS, NRPS, lanthipeptide-class-II, and NRPS-like domains, with an 80% similarity to the antimycin BGC. This genetic discovery aligns with our metabolomic analysis, where compound **4**, as well as compound **3**, were putatively annotated exclusively when CBMAI was elicited through co-culture. The production of this compound suggests a role as a defensive mechanism or in response to ecological cues. This integrated metabologenomic approach indicates CBMAI 1855′s ability to produce antimycin compounds under specific environmental conditions.

Recent research has identified the presence of both candicidin and antimycin BGCs in *Streptomyces albidoflavus*, highlighting their co-regulation [54,55]. The presence of these BGCs, akin to the surugamide BGCs documented in the *S. albidoflavus* genome, highlights their genomic association within the species. CBMAI 1855 harbors these three BGCs, with the candicidin and antimycin BGCs located adjacently (regions 44.1 and 45.1 as shown in Table 3). Candicidin biosynthesis in *S. albidoflavus* is controlled by cluster-situated regulators (CSRs) FscRI and FscRIV. These regulators activate gene expression within the same cluster, including two additional CSRs, FscRII and FscRIII. FscRI, which is known for activating the tetraene macrolide antifungal pimaricin biosynthesis in *Streptomyces natalensis,* also plays a role in this regulatory network [54,56,57,58]. This co-regulation mechanism suggests a sophisticated regulatory architecture that coordinates the production of multiple bioactive compounds in *S. albidoflavus*, potentially enhancing its adaptation to diverse environmental challenges [54].

CBMAI 1855 is a producer of multiple antifungal compounds, with various BGCs commonly found across its species and a wealth of unexplored biosynthetic potential that could be harnessed for the discovery of new antifungal agents. Selecting strains from extreme environments, such as CBMAI 1855, is a strategic approach for antifungal drug discovery, as its environments often drive the evolution of unique BGCs. Further investigation into these genomic regions, particularly regions 1.1, 40.2, and 41.1, is warranted to fully explore the strain’s potential in antimicrobial drug discovery. Techniques such as heterologous expression will be essential in future studies for elucidating the products and functions of these BGCs.

### 2.4. In Silico Predictions of Drug-Likeness and Toxicity

**Figure 5:**
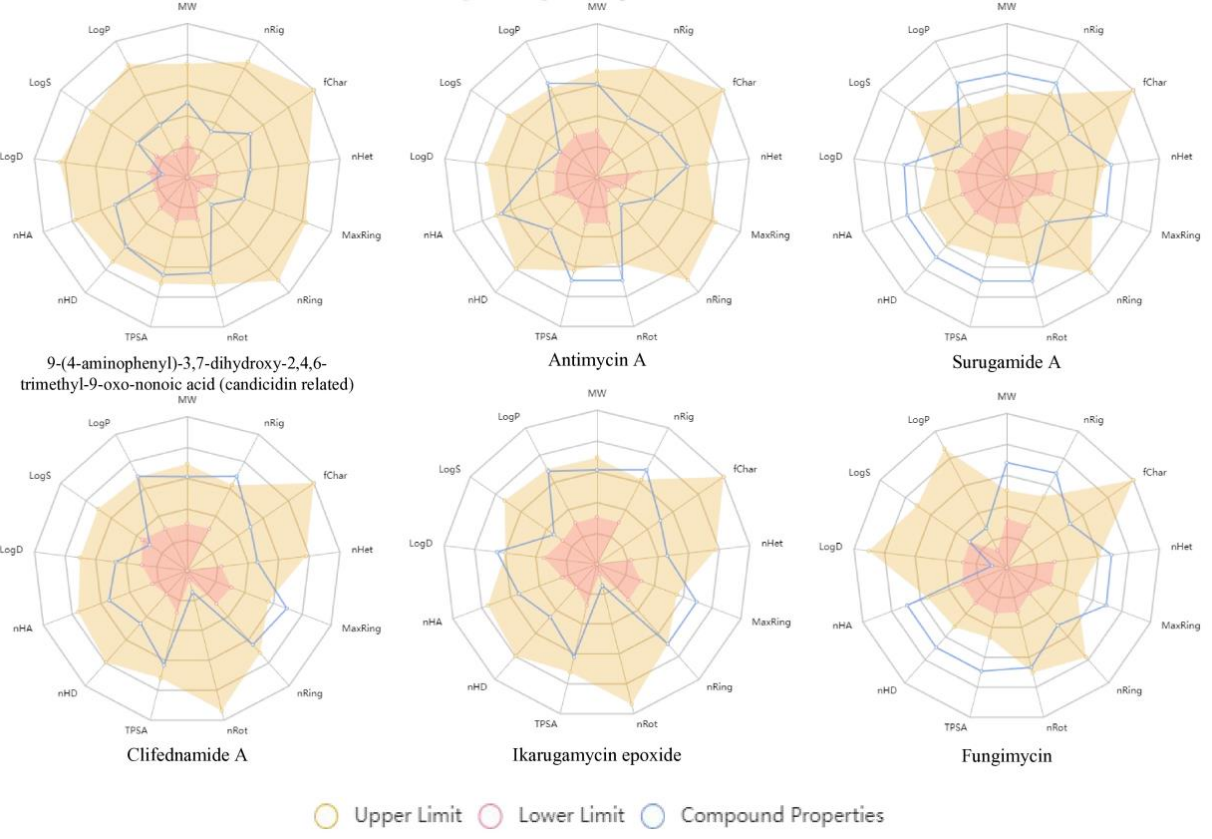
Radar chart made in ADMETlab2.0, visually representing the values of basic properties for each compound as a polygon. The chart depicts various physicochemical properties of six different molecules. The properties displayed are as follows: MW: molecular weight; nRig: number of rigid bonds; fChar: formal charge; nHet: number of heteroatoms; MaxRing: number of atoms in the biggest ring; nRing: number of rings; nRot: number of rotatable bonds; TPSA: topological polar surface area; nHD: number of hydrogen bond donors; nHA: number of hydrogen bond acceptors; LogD: LogP at physiological pH 7.4; logS: Log of the aqueous solubility and LogP: Log on the octanol/water partition coefficient. The upper limit and lower limit represent the acceptable or ideal ranges for drug-like properties. Values falling outside these boundaries may indicate potential issues with drug-likeness or bioavailability. The closer the polygon is to the outer boundary, the more the molecule fits within the desired range for drug-like properties. Large deviations or protrusions outside the open limit can highlight properties that may need optimization.

We evaluated the detected antifungal metabolites of CBMAI 1855 using Lipinski’s Rule of Five (Ro5) and additional pharmacokinetic parameters to predict their drug-likeness and potential for therapeutic use. Computational tools, including molecular property calculators and pharmacokinetic prediction software, were employed to generate these predictions based on the Canonical Simplified Molecular Input Line Entry System (SMILES) structures obtained from the GNPS annotation. The findings are detailed in Table 4 and visually represented in Figure 5. We have also evaluated the toxicity profiles of six antifungal metabolites using computational predictions. The toxicity parameters assessed included human hepatotoxicity, drug-induced liver injury (DILI), rat oral acute toxicity, skin sensitization, carcinogenicity, eye corrosion, eye irritation, and respiratory toxicity. The findings are detailed in Table 5.

Compound **1** is predicted to adhere to all the Ro5 criteria, with a molecular weight of 337.190, five hydrogen bond acceptors, and four hydrogen bond donors. The predictions suggest it may have good oral bioavailability potential with a LogP of 0.967 and aqueous solubility (LogS) of −2.702. This compound is also predicted to demonstrate high gastrointestinal absorption and not permeate the blood–brain barrier. It is not expected to inhibit cytochrome P450 enzymes, and it has a predicted bioavailability score of 0.56, indicating a potentially favorable profile for oral administration without significant metabolic issues or safety concerns.

Compound **4**, with a molecular weight of 492.210, is predicted to meet the Ro5 criteria for molecular weight and LogP (3.337). However, it has an excessive number of rotatable bonds [16], which may impact its conformational stability. Despite having nine hydrogen bond acceptors and four hydrogen bond donors, compound **4** is predicted to show low gastrointestinal absorption and a bioavailability score of 0.11. These predictions, combined with low solubility (LogS of −3.984), suggest limited potential as an orally administered drug. It is not expected to permeate the blood–brain barrier or inhibit cytochrome P450 enzymes.

Compound **8** is predicted to significantly violate Ro5 criteria due to its very high molecular weight (1094.590), ten rotatable bonds, and an excessive number of hydrogen bond donors [13] and acceptors [19]. Predictions indicate it may have low gastrointestinal absorption, as suggested by its bioavailability score, and poor aqueous solubility (LogS of −3.953). The lack of detailed pharmacokinetic information on cytochrome P450 inhibition and skin permeation further complicates its assessment for oral drug potential.

Compound **5** is predicted to exceed the acceptable limits for molecular weight (911.620) and LogP (5.136). It has sixteen rotatable bonds and nine hydrogen bond donors, which likely contribute to its predicted low gastrointestinal absorption (bioavailability score of 0.17). Despite its favorable number of hydrogen bond acceptors (nine), compound **5** is predicted to inhibit CYP3A4, suggesting potential drug–drug interactions. Its low solubility (LogS of −3.175) and lack of blood–brain barrier permeability are additional challenges to its drug-likeness.

Compound **6** is predicted to conform to Ro5 criteria with a molecular weight of 492.260, one rotatable bond, five hydrogen bond acceptors, and three hydrogen bond donors. It has a predicted LogP of 3.053 and may exhibit high gastrointestinal absorption, making it a viable candidate for oral administration. However, its very low aqueous solubility (LogS of −4.779) is a significant drawback. It is predicted to inhibit CYP3A4, indicating potential for drug–drug interactions, and has a bioavailability score of 0.56.

Compound **7** is predicted to satisfy Ro5 criteria with a molecular weight of 496.290, one rotatable bond, five hydrogen bond acceptors, and three hydrogen bond donors. Predictions indicate it may have high gastrointestinal absorption and a LogP of 2.893. Like compound **6**, it shows low solubility (LogS of −3.338) and is predicted to inhibit CYP3A4. Its bioavailability score of 0.55 indicates favorable drug-likeness, though the low solubility remains a challenge.

The evaluation of antifungal metabolites from CBMAI 1855 using Lipinski’s Rule of Five, pharmacokinetic parameters, and toxicity predictions provides a comprehensive prediction of their drug-likeness and safety profiles. The findings suggest that compound **1** exhibits the most promising characteristics, with high predicted gastrointestinal absorption, favorable solubility, and a relatively safe toxicity profile. Comparatively, compounds **4**, **8**, and **5** demonstrate multiple predicted violations of Lipinski’s rules and significant toxicity concerns, limiting their potential for oral administration. Compounds **6** and **7** show high gastrointestinal absorption and meet most drug-likeness criteria, although their low solubility and potential CYP3A4 inhibition pose challenges. These predictive analyses, while not definitive, provide valuable insights for prioritizing candidates for further experimental validation and potential optimization, as detailed in the tables and visually represented in Figure 5.

## 3. Discussion

To address the need for new antifungals, *Actinobacteria*, especially those isolated from extreme environments, are regarded as the main sources of antimicrobial drugs [59,60]. Their ability to produce a diverse array of bioactive compounds is attributed to their adaptation to harsh conditions, such as those found in Antarctica. This environment imposes extreme challenges, including low temperatures, high UV radiation, and nutrient scarcity, driving actinobacteria to evolve unique biochemical strategies for survival. These adaptations often result in the synthesis of metabolites with novel chemical structures and mechanisms of action, making them invaluable for drug discovery [31,32,61,62].

Informed by the WHO Fungal Priority Pathogens list [63], we selected public health critical isolates to address the most pressing threats. We have also focused on fungi with known resistance mechanisms, ensuring that our testing included pathogens with antifungal resistance profiles critical in medical and healthcare settings. This strategic choice marks one of the key differentiators of our study, as it reflects a targeted approach to identifying compounds with the potential to overcome fungal resistance.

The crude extract of CBMAI 1855 demonstrated antifungal activity against these pathogens, suggesting a broad-spectrum potential that includes strains resistant to conventional antifungal therapies. This finding is consistent with the notion that actinobacteria from extreme environments are a rich source of novel metabolites with multiple modes of action [31]. The broad-spectrum antifungal action of CBMAI 1855 could also be attributed to the production of multiple antifungal NPs by the strain, which may be presenting a synergistic effect. It is crucial for future studies to isolate and characterize all bioactive molecules produced by CBMAI 1855 and to examine their individual antifungal properties. Understanding the specific contributions of each compound and their potential synergistic interactions will be essential for fully harnessing the therapeutic potential of these NPs. Our work, which includes in silico predictions concerning the drug-likeness of annotated compounds and identifying genomic regions with the capacity to biosynthesize novel antifungal NPs, will inform decisions on prioritizing which compounds or BGCs are worth further investigation.

The MIC of 1.5 mg/mL observed in this study was consistent with the previous study involving *C. albicans* ATCC 10231 [33]. However, this MIC appears relatively high. We hypothesize that this elevated MIC may be attributed to the nature of CBMAI 1855, a marine microorganism that necessitates cultivation in artificial seawater. This cultivation process can result in the production of extracts containing a higher proportion of salts from the media, potentially generating a false mass and consequently reducing the relative concentration of bioactive molecules. This results in an overall higher mass than that of the pure bioactive compounds alone. Additionally, the high MIC could be attributed to the fact that we used monoculture, which can result in the production of fewer antifungal compounds due to the lack of elicitation [64,65]. It is also important to note that the tested sample was a crude extract, which contains a complex mixture of molecules [66], including components from the culture media. This complexity can affect the reliability of the MIC results due to the presence of non-bioactive substances that may contribute to the overall mass of the extract.

Microorganisms cultured alone often do not express the full range of their biosynthetic capabilities due to the silencing of certain genes under standard laboratory conditions. In contrast, co-culture can stimulate the production of specialized metabolites that are otherwise dormant, mimicking the natural competitive and interactive environments where microorganisms produce a diverse array of bioactive compounds as a survival mechanism [49,65,67,68]. The interaction between CBMAI 1855 and *A. flavus* likely triggered the activation of cryptic BGCs, leading to the production of unique antifungal compounds. This phenomenon is well-documented, as microbial interactions can induce the expression of silent BGCs, resulting in the biosynthesis of new metabolites with potential therapeutic applications [64,68]. Only through co-culture were we able to detect compounds **3** and **4**. Additionally, molecular networking revealed numerous unknown metabolites produced exclusively through co-culture, including novel metabolites within clusters of compounds of interest, such as those related to fungimycin, candicidin, and surugamides. It allows researchers to explore the hidden metabolic potential of microorganisms, facilitating the identification of novel compounds that could serve as effective antifungal agents. This method holds promise for overcoming the limitations of traditional monoculture approaches and enhancing the discovery pipeline for new drugs [49,64].

One of the significant challenges in antifungal drug discovery with NPs is the frequent rediscovery of known compounds. In our study of CBMAI 1855, we encountered known compounds. However, our findings highlight the importance of in silico predictions to assess their potential as therapeutic agents, offering critical insights into the drug’s likeness and potential toxicity [69]. Among the analyzed compounds **1**, **6**, and **7** emerge as a promising candidate due to their favorable pharmacokinetic predictions, while compounds **4** and **5** present significant hurdles. Additionally, compound **7** epoxide exhibits promising pharmacokinetic predictions and a relatively low toxicity profile, making it a candidate for further analysis with refinements to address moderate toxicity concerns.

While the findings derived from in silico predictions represent a limitation of this study, such predictive approaches are invaluable in drug discovery. Predicting the pharmacokinetics and toxicity profiles of compounds is crucial, as these characteristics often determine their viability in advancing through the antifungal drug development pipeline. By conducting in silico predictions, we can efficiently identify compounds with favorable pharmacokinetic and toxicity profiles, highlighting their promise as effective drugs. This process not only ensures that potentially viable candidates are not overlooked but also helps avoid the expenditure of time and resources on compounds that are unlikely to progress to lead status, thereby contributing to antifungal drug discovery. Future efforts can leverage the information obtained from these predictions to prioritize the isolation and study of compounds predicted to exhibit favorable characteristics. Ultimately, while acknowledging the limitations of predictions, they serve as a critical first step in guiding research and informing future experimental investigations, enhancing the efficiency of the drug discovery process [70,71,72].

CBMAI 1855 exhibits significant potential to produce antifungal metabolites, as evidenced by the identification of various BGCs encoding antifungal compounds within its genome. Some of these BGCs have been previously described in the literature as common to the species, while others are novel and unique to CBMAI 1855. Additionally, some BGCs identified are currently uncharacterized, representing unknown pathways with the potential to biosynthesize new antifungal compounds. In previous studies employing monoculture techniques, only compound **5** was annotated among the antifungal metabolites it is capable of producing [33]. However, by utilizing co-culture, we successfully activated and expressed most of these BGCs and correlated them with their metabolic products. Among the candicidin BGCs, the annotated metabolites include compounds **1**, **2**, and **3**. The antimycin BGC corresponds to the production of compound **4**. The three surugamides BGCs are associated with the production of compound **5**, surugamide D, and surugamide G. Additionally, the SGR PTMs BGC have been linked to compounds **6** and **7**. Our study has enabled the expression of a broader array of antifungal BGCs and provided insights into the potential efficacy of some compounds as therapeutic agents, highlighting the diverse antifungal biosynthetic potential of CBMAI 1855.

The differences between our findings and those reported by França et al. (2023) [33] can be attributed to several factors. While França’s study employed monoculture techniques, we utilized co-culture, which activated additional BGCs and enabled the detection of a broader array of metabolites. We also used an Orbitrap mass spectrometer for MS² data collection, known for its sensitivity, which likely contributed to capturing a wider range of bioactive compounds. Our extraction process was distinct, as we selectively cut sections of agar where metabolite diffusion was anticipated, optimizing recovery. Additionally, experimenting with smaller volumes allowed us greater control, while using a different solvent and SPE cartridges may have enhanced the retention of bioactive metabolites. Lastly, the use of different methodologies for processing the extracts can result in slight differences in compound detection. These combined factors help explain the variation in our results using the same strain.

Regions 1.1 (an NRPS linked to diisonitrile antibiotics), 40.2, and 41.1 are highlighted for further exploration. Regions 40.2 and 41.1 exhibit low similarity to the candicidin and rosamicin BGCs, respectively. The positioning of these regions near the naringenin and candicidin/antimycin BGCs enhances their potential significance. The low similarity of these BGCs to known clusters suggests the presence of potentially novel biosynthetic pathways, as we have yet to discover the compounds derived from these regions. Given the location and potential of these gene clusters, further investigation is warranted to characterize novel therapeutic agents against fungal infections. These regions are noteworthy for their potential to produce structurally diverse metabolites with enhanced biological activities.

To gain deeper insights into the prevalence of different BGCs within *S. albidoflavus* clades from regions outside Antarctica, we analyzed previously reported genome mining data from various isolates. Du et al. (2022) [73] examined strain St-220, which was isolated from the rhizosphere of *Salvia miltiorrhiza* in China. Their analysis identified several antifungal BGCs that are also present in our Antarctic strain CBMAI 1855, including diisonitrile antibiotic SF2768, SGR PTMs, surugamides, antimycin, and candicidin. St-220 possesses a BGC for fredericamycin A, absent in CBMAI 1855. This indicates that while both strains share some common BGCs, they also harbor unique BGCs that could encode distinct antimicrobial compounds. Diabankana et al. (2023) [74] analyzed the genome of strain MGMM6, isolated from the rhizosphere soil of spring wheat in Russia. This strain also possesses BGCs for SGR PTMs, candicidin, antimycin, and surugamides, along with additional BGCs that are not found in CBMAI 1855, such as fredericamycin A, dudomycin A, and minimycin. Kunova et al. (2021) [75] analyzed two strains, DEF1AK and DEF147AK, isolated from plant roots in Italy, alongside the type strain DSM 40455 T. Both Italian strains contain BGCs for SGR PTMs, antimycin, surugamides, and concanamycin A, which is not present in other analyzed strains. Strain ACT77, isolated from soil under agricultural management in Brazil by Pylro et al. (2019) [76], possesses BGCs for SGR PTMs and surugamides. However, the antimycin BGC was not found in this strain, although it is present in other isolates. The candicidin BGC was absent in DEF1AK but detected in other strains.

Most *S. albidoflavus* strains analyzed exhibit BGCs familiar to CBMAI 1855, such as SGR PTMs, surugamides, and antimycin (though it can be absent in some strains), as well as candicidin (which can also be absent in certain strains). Additionally, all strains contain unique cryptic BGCs that vary among isolates. CBMAI 1855 contains cryptic BGCs, such as regions 40.2 and 41.1. For instance, the Italian strains possess the concanamycin A BGC. In contrast, strains MGMM6 and St-220 harbor the fredericamycin A BGC, which is absent in CBMAI 1855 and the Italian strains DEF1AK, DEF147AK, and the strain DSM 40455 T. This analysis highlights the diversity of biosynthetic potential within *S. albidoflavus* strains, revealing both conserved and unique features across different geographical regions.

Beyond identifying known molecules, our study highlights CBMAI 1855’s potential as a source of novel antifungal compounds. While we detected known compounds, the genome of CBMAI 1855 contains regions, such as the candicidin BGC, that may produce novel analogs. Notably, we did not annotate the most described candicidins, such as candicidin D, A3, or A1. Instead, using the FBMN tool on GNPS, we successfully annotated compounds resembling candicidin I and candicidin IV, which have limited information in the literature and databases. The lack of comprehensive molecular data constrained subsequent computational predictions of drug-likeness and toxicity due to incomplete chemical information within GNPS and a scarcity of detailed references on these specific candicidin analogs. The presence of many unannotated molecules in the molecular networking clusters suggests that CBMAI 1855 harbors unique bioactive compounds. GNPS, the primary platform for molecular networking, organizes mass spectrometry data by grouping compounds that fragment similarly. In our study, the candicidin analog cluster included fungimycin and numerous unannotated molecules, indicating the presence of potentially novel polyene macrolides compounds.

GNPS is an essential tool in NP research for drug discovery, consolidating data from major databases and facilitating the identification of NP through molecular networking. When compounds in a cluster cannot be annotated using GNPS and other databases, it suggests that these compounds are novel. This capability is particularly valuable for uncovering new bioactive molecules that may have therapeutic potential. Our study demonstrates that, while rediscovery is a common challenge, the use of advanced analytical techniques and platforms like GNPS can reveal the hidden potential of microbial sources like CBMAI 1855, offering new avenues for antifungal drug discovery.

Our integrated metabolomic and genomic approach has unveiled CBMAI 1855 as a reservoir of diverse antifungal metabolites. The combination of genome mining and metabolomic profiling has identified cryptic BGCs capable of producing novel antifungal agents. Each of these cryptic BGCs warrants further exploration through heterologous expression, promising insights into their bioactive properties and therapeutic applications. This holistic approach enhances our understanding of CBMAI 1855′s chemical diversity, positioning the strain as a valuable resource for future drug discovery endeavors.

CBMAI 1855, a strain of *Streptomyces albidoflavus* isolated from Antarctica, emerges as a prolific producer of multiple antifungal compounds. The extreme environmental conditions of its habitat likely contribute to the evolution of its extensive arsenal of antifungal metabolites, crucial for microbial survival and adaptation. *Streptomyces albidoflavus* is referred to in the literature for its effective biological control of fungi in agricultural settings [73,77], demonstrating its ecological significance. Our study not only reaffirms these capabilities but also emphasizes the untapped potential of strains isolated from extreme environments, such as Antarctica, in pharmaceutical research. CBMAI 1855 exemplifies this potential, offering a promising pathway toward the discovery and development of novel antifungal therapies with significant implications for human health and agricultural sustainability.

## 4. Materials and Methods

### 4.1. Strains

#### 4.1.1. *Streptomyces albidoflavus* CBMAI 1855

*Streptomyces albidoflavus* CBMAI 1855, stored in an ultra freezer at −80 °C, is cataloged in the Coleção Brasileira de Microorganismos de Ambiente e Indústria (CBMAI) (https://cbmai.cpqba.unicamp.br/ (accessed on 24 August 2024) housed at the Divisão de Recursos Microbianos [CPQBA/University of Campinas (Unicamp)]. The isolate was reactivated by inoculation onto Nutrient Agar (NA, Difco, Detroit, MI, USA) supplemented with artificial seawater (ASW). The ASW composition included KBr (0.1 g/L), NaCl (23.48 g/L), MgCl_2_·6H_2_O (10.61 g/L), CaCl_2_·2H_2_O (1.47 g/L), KCl (0.66 g/L), SrCl_2_·6H_2_O (0.04 g/L), Na_2_SO_4_ (3.92 g/L), NaHCO_3_ (0.19 g/L), and H_3_BO_3_ (0.03 g/L). The inoculated plates were incubated at 28 °C for five days. Post incubation, bacterial colonies were examined for purity and used for further experimental analyses.

#### 4.1.2. Fungal Pathogens

The fungal pathogen isolates utilized in this study were sourced from the University of Campinas Clinical Hospital (HC-Unicamp), a tertiary referral hospital located in Campinas (https://www.hc.unicamp.br (accessed on 24 August 2024)). These samples are maintained by the Fungal Investigation Laboratory (LIF) culture collection at the School of Medical Sciences, University of Campinas. The isolates are preserved in water at room temperature using the Castellani method [24].

The strains were selected from the LIF collection. Firstly, we prioritized the inclusion of standard ATCC (American Type Culture Collection) strains, which are widely recognized and used internationally for susceptibility testing and quality control references. We prioritized genera and species listed in the World Health Organization (WHO) Fungal Priority Pathogens List to guide research (FPPL) [25], which was created in 2022 and includes fungi categorized as critical, high, and medium priority. This list highlights fungi that pose significant health threats to guide research by drawing attention to pathogens that are becoming more common, resistant to treatment, and lethal. Our focus was on genera and species listed as Critical Priority and High Priority, with additional inclusion from the Medium Priority list. We also included a dermatophyte to assess bioactivity against fungi that might be suitable for topical treatment if the molecules in question have limited bioavailability or high toxicity in future assays.

Additionally, we included LIF collection isolates of *C. albicans* and *A. fumigatus* with resistance mutations. *C. albicans* and *A. fumigatus* are among the most clinically significant yeasts and molds causing fungal infections [78], emphasizing the relevance of studying their response to potential antifungal compounds. This approach can provide valuable insights into the target of bioactive molecules from the extract, particularly if a molecule proves ineffective against strains with specific mutations. We also considered intrinsic resistances, such as those found in *Aspergillus* species and *Candida krusei*, both intrinsically resistant to FCZ [79,80], as well as *Aspergillus terreus*, which is genetically resistant to FCZ and the polyene AMB [81]. Isolates of the “super yeast” *Candida auris*, exhibiting multi-susceptible and AMB resistance, were also included. This diverse range of isolates with different resistance profiles allows for a comprehensive exploration of the efficacy of the NPs produced by CBMAI 1855. The fungal isolates included were as follows:

Control strains—American Type Culture Collection (ATCC) standard strains:

- *Candida albicans* (ATCC 90028);
- *Candida parapsilosis* (ATCC 22019);
- *Candida krusei* (ATCC 6258);
- *Aspergillus fumigatus* (ATCC 204305);
- *Aspergillus flavus* (ATCC 204304);
- *Cryptococcus neoformans* (ATCC 90113);
- *Cryptococcus gattii* (ATCC 56990);
- *Trichophyton mentagrophytes* (ATCC 9533).

The isolates from patients at HC-Unicamp were as follows:

- LIF 12560 (*C. albicans*): The resistance mechanism involves substitutions at amino acids E116D, T128K, E266D, and A298V in the ERG11 gene [82];
- LIF E-10 (*C. albicans*): The resistance mechanism involves substitutions G448V and G464S in the ERG11 gene [82];
- LIF 2552-4.9 (*A. fumigatus*): The resistance mechanism is characterized by the CYP51A TR34/L98H/S297T/F495I mutations [83];
- LIF 2444.6 (*A. fumigatus*): The resistance mechanism is characterized by the CYP51A TR34/L98H/S297T/F495I mutation [83];
- LIF 263-e (*A. fumigatus*): The resistance mechanism is characterized by the CYP51A TR46/F495I mutation [84];
- LIF 1889 (*A. fumigatus*): No mutations were observed in the CYP51A gene, and the resistance mechanism remains still unknown (unpublished data);
- LIF 2328 (*A. fumigatus*): No mutations were observed in the CYP51A gene, and the resistance mechanism remains still unknown [84];
- LIF 2486 (*A. terreus*);
- LIF 16607 (*C. auris*): Sensitive to all antifungal agents tested (unpublished data);
- LIF 16615 (*C. auris*): Exhibits resistance to amphotericin B (unpublished data);
- LIF 1455 (*Rhizopus oryzae*).

For reactivation, the strains were inoculated onto Sabouraud Dextrose Agar (SDA, Difco Laboratories, Detroit, MI, USA) and incubated under optimal growth conditions specific to each group (filamentous fungi and yeast). Following incubation, the isolates were inspected for purity and subsequently used for bioactivity testing.

### 4.2. Bioactivity Assays

#### 4.2.1. Antagonism Test (Co-Culture)

To evaluate the bioactivity of secondary metabolites produced by CBMAI 1855 against fungi, a dual culture assay was performed, as adapted from Chen et al., 2018 [85]. CBMAI 1855 was streaked vertically on opposite sides of a petri dish containing MHA. The plates were then incubated at 28 °C for five days to allow the bacterial metabolites to diffuse into the agar. After this incubation period, a fungal inoculum of *Aspergillus flavus* ATCC 204304 was placed in the center of the same plate. The co-culture was then incubated for an additional six days at 28 °C to allow potential antagonistic interactions to develop. A control plate containing only the fungal inoculum (without the bacterium) was also incubated under the same conditions.

*Aspergillus flavus* ATCC 204304 was selected for co-culture due to its designation as a reference strain by international guidelines, including those from the Clinical and Laboratory Standards Institute (CLSI) and the European Committee on Antimicrobial Susceptibility Testing (EUCAST) [86,87,88]. Commonly used for quality control and susceptibility testing in fungi across clinical laboratories, this strain enhances the reliability and interpretability of our antifungal susceptibility findings. It grows relatively fast, making it ideal for timely testing, while its consistent growth parameters ensure the reproducibility of experimental results and clear observation of inhibition zones, which are essential for our analysis.

#### 4.2.2. Broth Microdilution

For broth microdilution assays, CBMAI 1855 extracts were initially produced following the method described by De França et al. (2022) [33]. A starter culture (50 mL) served as the basis for scaling up to larger volume cultures (100 mL, 500 mL) of Nutrient Broth (NB, Oxoid™) medium supplemented with ASW. These cultures were then incubated at 28 °C for 30 days under stationary conditions. In the production of crude extracts, a negative control, representing the culture media without bacterial growth, was included.

Following the incubation period, ethyl acetate was added to the liquid culture at a 1:1 ratio, and the mixture of medium and solvent was vigorously mixed for 5 min. The extracts were obtained using the liquid–liquid extraction method, and the organic phases were concentrated under vacuum using a Rotary evaporator (Buchi^®^ R-215, Flawil, Switzerland) at 40 °C and 80 rpm. A negative control, representing the culture media without bacterial growth, was included in the production of crude extracts.

The antifungal activity of the crude extract was evaluated to determine the MIC and MFC through microdilution assays following the guidelines set by the CLSI, utilizing the M27-A3 [86] document for yeasts and the M38-A2 [87] document for filamentous fungi. The MIC represents the lowest concentration of an antimicrobial agent that inhibits the visible growth of a microorganism. In contrast, the MFC represents the lowest concentration of the extract that kills the initial inoculum [33]. The crude extracts of CBMAI 1855 were solubilized in RPMI broth with 2% DMSO and assayed at concentrations ranging from 0.0078 to 3.0 mg/mL. To assess the possibility of contamination, crude extracts were incubated without microorganism inoculation, and the viability of cells was confirmed during the test. RPMI served as the negative control in all experiments, conducted in triplicate. To determine the MFC, after the MIC reading, ten μL from each well that did not exhibit growth in the broth microdilution plate were seeded onto SDA plates and incubated at 35 °C for 24–48 h to confirm the absence of fungal growth.

Following the M27-A3 document for yeasts and the M38-A2 document for filamentous fungi, we also conducted susceptibility testing of the same strains against commercial antifungal drugs to compare the MIC values of the CBMAI 1855 extract with the MIC and MEC values of the commercial antifungals. It is important to note that MEC is specifically reported for echinocandin drugs (micafungin and caspofungin), while MIC values are provided for the other commercial antifungals. Commercial antifungals also served as positive controls in the broth microdilution tests.

### 4.3. Untargeted Metabolomic Analysis

#### 4.3.1. Sample Preparation

For high-resolution MS/MS analysis, we extracted NPs from the co-culture of CBMAI 1855 and *A. flavus* ATCC 204304—a well-established and reproducible reference strain commonly employed in quality control and susceptibility testing. The co-culture approach was selected for metabolomic analysis due to its potential to induce the production of a broader spectrum of secondary metabolites that may not be synthesized when CBMAI 1855 is grown in monoculture. Following the co-culture incubation period, as described in Section 4.2.1, inhibition zones—where diffused bacterial NPs successfully inhibited fungal growth—were excised from the agar. These zones were used for subsequent metabolite extraction and analysis, following a modified version of the protocol by Maimone et al., 2021 [49]. In brief, the excised inhibition zones were first lyophilized. The dried material was then extracted using a CH_2_Cl_2_-MeOH-H_2_O (64:36:8, *v*/*v*/*v*) mixture, applying a ratio of 200 mL of solvent per 300 g of dry material. The extraction was performed using an Ultra-Turrax homogenizer IKA^®^, Staufen, Germany) for 1 min to ensure efficient recovery of the NPs. The crude extracts were filtered using glass wool to remove any residual agar debris and subsequently concentrated under reduced pressure using a rotary evaporator (Buchi® R-215, Flawil, Switzerland). Further purification involved resuspending the concentrated extract in ultrapure water, followed by fractionation using C18 SPE cartridges (Welcrom^®^, Jinan, China). A gradient elution was performed with 10 mL of H_2_O, 10 mL of H_2_O/MeOH (1:1 *v*/*v*), and 10 mL of MeOH. The first fraction, which contained only water, was discarded, and the remaining fractions were pooled, concentrated to a final volume of 1.5 mL, and prepared for MS/MS data acquisition. As controls, we included monocultures of CBMAI 1855 and *A. flavus* ATCC 204304, along with sterile MHA plates as blanks. All experimental treatments were performed in triplicate to ensure reproducibility.

#### 4.3.2. Data Acquisition

The UPLC-MS/MS data were acquired using a Thermo Scientific Q-Exactive Orbitrap mass spectrometer (Thermo Fisher Scientific, Waltham, MA, USA), operating in positive ion mode. Sample analyses were performed in Full Scan/Data-Dependent MS/MS (Top N) mode, which involved an MS survey scan ranging from 100 to 1500 Da, followed by MS/MS scans for the five most intense ions. The collision energy was set to a gradient from 20 to 40 V. The DESI source conditions were as follows: sheath gas flow rate at 35, auxiliary gas flow rate at 10, spray voltage at 10 kV, capillary temperature at 300 °C, S-lens RF level at 50.0, auxiliary gas heater temperature at 50 °C, and *m*/*z* range from 100.0 to 1500.0. Data acquisition and analysis were conducted using Xcalibur software (version 3.0.63, Thermo Fisher Scientific, Waltham, MA, USA). For chromatographic separations, an Acquity UPLC BEH C18 column (1.7 μm, 2.1 × 50 mm, Waters, Milford, MA, USA) was used as the stationary phase. The mobile phase consisted of a gradient of (A) ultrapure water with 0.1% formic acid and (B) acetonitrile with 0.1% formic acid. The gradient elution program was as follows: 90%A:10%B for 9 min, 50%A:50%B for 6 min, 2%A:98%B for 5 min, and re-equilibration at 90%A:10%B for 4 min, totaling 24 min of analysis. The flow rate was set at 0.3 mL/min. The column temperature was maintained at 40 °C, and the injection volume for each sample was 1 μL.

#### 4.3.3. Molecular Network and Metabolite Annotation

Raw data files from the triplicates of co-culture, CBMAI 1855 and *A. flavus* ATCC 204304, solvent, and media blanks were converted to .mzML format using MSConvert software version 3.0 (http://proteowizard.sourceforge.net (accessed on 24 August 2024)). These files were then uploaded to Global Natural Product Social Molecular Networking (GNPS) via WinSCP (version 6.3.3) and analyzed using the Classical Molecular Networking (CMN) tool within the GNPS platform (http://gnps.ucsd.edu (accessed on 24 August 2024)). For the analysis, data were filtered to exclude all MS/MS fragment ions within ±17 Da of the precursor *m*/*z*. MS/MS spectra were window-filtered by selecting only the top six fragment ions within a ±50 Da window across the spectrum. The precursor and fragment ion mass tolerances were set to 0.02 Da. Subsequently, a molecular network was constructed, retaining edges with a cosine score above 0.65 and more than four matched peaks. Edges between two nodes were kept only if each node was among the top 10 most similar nodes. The maximum size of a molecular family was capped at 100, and the lowest-scoring edges were removed to maintain this limit. The spectra within the network were then matched against GNPS’ spectral libraries, applying the same filtering criteria for the input data. Matches between network and library spectra required a score above 0.7 and at least six matched peaks. To establish the confidence level achieved in metabolite annotation, we adopted the accuracy standards set by the Metabolomics Standards Initiative (MSI) [89]. The MSI, an international community dedicated to standardizing metabolomics data reporting to enhance information exchange, proposes four levels of metabolite identification. The resulting molecular networks were visualized using Cytoscape 3.10.2, and known metabolites were annotated by comparing their mass and fragmentation patterns with those in the GNPS spectral libraries and cross-referencing with published data. Our job can be found at https://gnps.ucsd.edu/ProteoSAFe/status.jsp?task=5f4efd01adb648eb9c456730445ebdd2 (accessed on 24 August 2024). Interesting spectra in the network were searched in GNPS and other online spectral libraries. Additionally, further analysis using the Dereplicator+ tools on the GNPS platform was conducted (job link: https://gnps.ucsd.edu/ProteoSAFe/status.jsp?task=3d15a5e50a994a159e5389211ba216b0 (accessed on 24 August 2024)). The best formulas were selected and searched in the Natural Products Atlas database [90], focusing on *Streptomyces* as the source genera.

#### 4.3.4. Statistical Analysis

For statistical analysis, the mz.xml data were imported into MZmine 4.0.8 software and processed using the following steps and parameters. Mass detection: a noise level of 1000 for MS1 and 900 for MS2 was applied. Chromatogram builder: settings included a minimum time span of 0.15 min, a minimum peak height of 1000, and an *m*/*z* tolerance of 0.02 Da. Chromatogram deconvolution: the ‘local minimum search’ algorithm was used with a retention time range of 0.1 min, a chromatographic threshold of 50%, a minimum relative height of 30%, a minimum absolute height of 1000, a minimum peak top/edge ratio of 1, and a peak duration range of 0.05–1.5 min. Isotopic peaks grouper: an *m*/*z* tolerance of 0.02 Da and a retention time tolerance of 0.1 min were used, with the most intense peaks chosen as representatives. Alignment: the ‘join aligner’ algorithm was employed with an *m*/*z* tolerance of 0.02 Da (weight: 75%) and a retention time tolerance of 0.1 min (weight: 25%). Feature list filtering: the feature list blank subtraction and feature list row filter, for only the peaks present in at least two samples and those with MS2 scans, were retained. The generated peak list was exported as a .csv file containing the peak areas for all detected scans. These data were then used to construct Feature-based Molecular Networking (FBMN) [job: https://gnps.ucsd.edu/ProteoSAFe/status.jsp?task=b240a4e0319640f58fc472b721018d19 (accessed on 24 August 2024)] and were also analyzed statistically using the MetaboAnalyst tool version 6.0 [91]. In MetaboAnalyst, the data were first filtered by the interquartile range, normalized by sum, and log-transformed. PCA and a hierarchical clustering dendrogram based on the Euclidean distance measure and Ward’s clustering algorithm were generated. Additionally, a hierarchical clustering heatmap was constructed to visualize differential metabolites of importance in co-culture, bacterial monoculture, and fungal monoculture samples. Parameters for the heatmap included Pearson’s correlation for distance measurement and complete linkage for clustering, which were performed using the hclust function in the stats package.

### 4.4. Antifungal Genome Mining

The genetic potential of CBMAI 1855 was analyzed using antiSMASH version 7.0 [92], focusing specifically on BGCs relevant to antifungal NP production. The genome sequence of CBMAI 1855 is available in GenBank under the accession number JADILI000000000. AntiSMASH 7.0 utilized these genomic data with default parameters including ClusterBlast, KnownClusterBlast, SubClusterBlast, MIB-iG cluster comparison, ActiveSiteFinder, RREFinder, Cluster Pfam analysis, Pfam-based GO term annotation, TIGRFam analysis, and TFBS analysis.

### 4.5. In Silico Predictions of Drug-Likeness and Toxicity

The drug-likeness properties and toxicity predictions of the annotated metabolites with previously described antifungal activity were assessed using the SwissADME tool (http://www.swissadme.ch/ (accessed on 24 August 2024)), which is an online tool continuously updated, and the ADMETlab version 2.0 server (https://admetmesh.scbdd.com/ (accessed on 24 August 2024)) [93,94]. SMILES structures of the metabolites were inputted into the servers. The analysis was based on Lipinski’s Rule of Five (Ro5), which includes four key parameters: molecular weight (MW ≤ 500), consensus LogP value (LogP ≤ 5), number of hydrogen bond donors (HBD ≤ 5), and number of hydrogen bond acceptors (HBA ≤ 10). These parameters help to predict the drug-likeness of compounds, although meeting these criteria alone does not guarantee oral activity. Additional descriptors, such as the number of rotatable bonds (RB ≤ 10), were also considered to provide a more comprehensive assessment of the compounds’ likelihood of being biologically active. Toxicity analysis included predictions of potential toxicological properties, such as mutagenicity, carcinogenicity, hepatotoxicity, and organ toxicity. The ADMETlab 2.0 server provided a detailed profile of each compound’s toxicological endpoints, enabling the identification of any potential toxic effects that could impact their suitability as drug candidates [94,95].

## 5. Conclusions

Overall, this study demonstrates that CBMAI 1855 is a prolific producer of multiple antifungal agents, with the co-culture approach proving instrumental in unlocking this potential for drug discovery. The strain’s capacity to synthesize novel antifungal compounds highlights its significance as a promising source of new therapeutics. Future studies should also aim to heterologously express the cryptic BGCs to elucidate their products fully and exploit the strain’s biosynthetic capabilities to their fullest extent. Additionally, integrating advanced dereplication methods with genome mining proved to be crucial for identifying and harnessing these bioactive metabolites effectively.

## Figures and Tables

**Table 1:** Broth microdilution assays of ethyl acetate extract, and MIC values of fungal isolates to commercial antifungal drugs.

Strain	Susceptibility to Ethyl Acetate Crude Extract (mg/mL)		Susceptibility Profile to Commercial Drugs (MIC/MEC in µg/mL)								
			MCF	CPF	AMB	5-FC	FCZ	VRC	ITZ	MCZ	TER
*C. albicans* (ATCC 90028)	MIC	1.5	0.5	0.12	1	-	1	0.03	0.12	0.5	-
	MFC	1.5									
*C. parapsilosis* (ATCC 22019)	MIC	1.5	0.25	1	1	-	1	0.25	0.12	0.5	-
	MFC	1.5									
*C. krusei* (ATCC 6258)	MIC	1.5	≤0.015	0.25	1	-	-	0.25	0.12	0.25	-
	MFC	1.5									
*A. fumigatus* (ATCC 204305)	MIC	1.5	≤0.015	0.25	1	-	>64	0.5	0.5	1	-
	MFC	1.5									
*A. flavus* (ATCC 204304)	MIC	1.5	≤0.015	0.25	1	-	>64	0.25	0.5	1	-
	MFC	1.5									
*C. neoformans* (ATCC 90113)	MIC	1.5	-	-	0.5	1	2	0.12	0.06	-	-
	MFC	3.0									
*C. gattii* (ATCC 56990)	MIC	1.5	-	-	0.5	0.5	2	0.015	0.12	-	-
	MFC	3.0									
*T. mentagrophytes* (ATCC 9533)	MIC	1.5	-	-	-	-	-	-	0.125	-	0.03
	MFC	1.5									
LIF 12560	MIC	1.5	-	-	0.25	<0.12	8	0.25	0.12	-	-
	MFC	1.5									
LIF E-10	MIC	1.5	-	-	0.25	<0.12	64	0.25	1	-	-
	MFC	1.5									
LIF 2552-4.9	MIC	1.5	≤0.015	0.25	1	-	-	4	>8	>16	-
	MFC	1.5									
LIF 2444.6	MIC	1.5	≤0.015	0.25	1	-	-	2	>8	>16	-
	MFC	1.5									
LIF 263-e	MIC	1.5	≤0.015	0.125	1	-	-	4	>8	>16	-
	MFC	1.5									
LIF 1889	MIC	1.5	0.03	0.25	1	-	-	8	4	>16	-
	MFC	1.5									
LIF 2328	MIC	1.5	≤0.015	0.25	2	-	-	1	0.5	-	-
	MFC	1.5									
LIF 2486	MIC	1.5	≤0.015	0.25	1	-	-	2	>8	>16	-
	MFC	1.5									
LIF 16606-1	MIC	1.5	0.12	0.5	0.5	0.12	0.5	0.03	0.03	-	-
	MFC	1.5									
LIF 16615	MIC	1.5	0.07	0.25	8	-	0.03	0.07	0.07	-	-
	MFC	1.5									
LIF 1455	MIC	1.5	≥ 16	-	0.5	>64	>64	>8	>8	1	>16
	MFC	1.5									

MIC = minimum inhibitory concentration, MFC = Minimal Fungicide Concentration, MEC = minimal effective concentration; MEC was defined for echinocandins and MIC was defined for the other drug classes and the extract. AMB = amphotericin B, FCZ = fluconazole, MCF = micafungin, ITZ = itraconazole, VOR = voriconazole, 5FC = 5-flucytosine, MCZ = miconazole, TER = terbinafrine.

**Table 2:** Putatively annotated metabolites with known or potential antifungal bioactivity.

Compound	#	Annotation	Molecular Formula	Cosine Score	Spec MZ	MetMass	NP Pathway	NP Superclass	NP Class
9-(4-aminophenyl)-3,7-dihydroxy-2,4,6-trimethyl-9-oxo-nonoic acid (candicidin related)	1	GNPS	C_18_H_27_NO_5_	0.88	364.21	338.20	Polyketides (PKS)	Linear PKS	Open-chain PKS
Candicidin I	2	GNPS (Analog library hit)	-	0.74	546.29	547.28	-	-	-
Candicidin IV	3	GNPS (Analog library hit)	-	0.71	557.31	556.28	-	-	-
Antimycin A	4	DEREPLICATOR+	C_28_H_40_N_2_O_9_	-	507.234	506.23	-	-	-
Surugamide A	5	GNPS	C_48_H_81_N_9_O_8_	0.91	912.62	911.62	Amino acids and Peptides	Oligopeptides	Cyclic peptides
Clifednamide A	6	GNPS	C_29_H_36_N_2_O_5_	0.76	495.28	493.27	Amino acids and Peptides	Macrolides	Macrocyclic tetramic acids (PTM)
Ikarugamycin epoxide	7	GNPS	C_29_H_38_N_2_O_5_	0.79	497.30	495.29	Amino acids and Peptides	Macrolides	PTM
Fungimycin	8	DEREPLICATOR+	C_59_H_86_N_2_O_17_	-	1095.6	1094.59	-	-	-

**Table 3:** Summary of BGCs in CBMAI 1855 likely producing antifungal compounds.

Region	Type	From	To	Most Similar Known Cluster		Similarity
Region 1.1	NRPS	164.68	209.1	diisonitrile antibiotic SF2768	NRP	66%
**Region 1.3**	**T1PKS, NRPS**	**423.35**	**472.76**	**SGR PTMs**	**NRP + PKS**	**100%**
**Region 24.1**	**NRPS**	**5.317**	**35.206**	**surugamide A/surugamide D**	**NRP**	**42%**
**Region 25.1**	**NRPS**	**1**	**41.377**	**surugamide A/surugamide D**	**NRP**	**19%**
**Region 27.1**	**NRPS, LAP**	**1**	**34.45**	**surugamide A/surugamide D**	**NRP**	**61%**
Region 40.1	T3PKS	1.86	42.957	naringenin	T3PKS	100%
Region 40.2	T1PKS	47.893	101.94	candicidin	NRP + PKS	33%
Region 41.1	T1PKS	1	31.408	rosamicin	PKS	26%
**Region 44.1**	**T1PKS, NRPS-like**	**1**	**59.019**	**candicidin**	**NRP + PKS**	**90%**
**Region 45.1**	**NRPS, T1PKS, LAPclass-II, NRPS-like**	**1**	**74.671**	**antimycin**	**NRP: Cyclic depsipeptide + T3PKS**	**80%**

Legend: Region = the region number in the genome assigned by antiSMASH. Type = the product types as detected by antiSMASH: NRPS (Non-ribosomal peptide synthetase), T1PKS (Type I polyketide synthase), T3PKS (Type III polyketide synthase), LAP (Lantibiotic and other peptide synthetase), NRPS-like (Non-ribosomal peptide synthetase-like), LAPclass-II (Class II lanthipeptide). From, To: the location of the region in nucleotides. Most similar known cluster: the closest compound from the MiBIG database, along with its type. Similarity: a percentage of genes within the closest known compound that have a significant BLAST hit to genes within the current region. Bolded regions indicate expressed biosynthetic BGCs, which are the BGCs where we putatively annotated the respective metabolites produced.

**Table 4:** Lipinski and additional parameters to examine drug-likeness of the antifungal metabolites.

Compound Annotated	Physicochemical Properties						Pharmacokinetics				
	Molecular Weight	N° Rotatable Bonds	N° H-Bond Acceptors	Nº H-Bond Donors	Aqueous Solubility (LogS)	Octanol/Water Partition Coeffifient (LogP)	Gastrointestinal Absorption	Blood-Brain Barrier Permeability	Metabolism (Cytochrome P450 Inhibition)	Skin Permeation (Log Kp) [cm/s]	Bioavailability Score
9-(4-aminophenyl)-3,7-dihydroxy-2,4,6-trimethyl-9-oxo-nonoic acid (candicidin related)	337.190	9	5	4	−2.702	0.967	High	No	No	−7.19	0.56
Antimycin A	492.210	16	9	4	−3.984	3.337	Low	No	No	−6.76	0.11
Fungimycin	1094.590	10	19	13	−3.953	0.649	Low	No	No information	No information	No information
Surugamide A	911.620	16	9	9	−3.175	5.136	Low	No	Yes (CYP3A4)	−7.65	0.17
Clifednamide A	492.260	1	5	3	−4.779	3.053	High	No	Yes (CYP3A4)	−6.95	0.56
Ikarugamycin epoxide	496.290	1	5	3	−3.338	2.893	High	No	Yes (CYP3A4)	−6.91	0.55

**Table 5:** Toxicity predictions of the antifungal metabolites.

Compound Annotated	Toxicity							
	Human Hepatotoxicity	Drug-Induced Liver Injury	Rat Oral Acute Toxicity	Skin Sensitization	Carcinogenicity	Eye Corrosion	Eye Irritation	Respiratory Toxicity
9-(4-aminophenyl)-3,7-dihydroxy-2,4,6-trimethyl-9-oxo-nonoic acid (candicidin related)	--	+++	-	---	---	---	---	-
Antimycin A	++	+++	---	--	---	---	---	---
Fungimycin	+++	---	---	+++	+	---	---	+++
Surugamide A	+++	---	++	---	---	---	---	---
Clifednamide A	-	+++	-	--	--	---	---	++
Ikarugamycin epoxide	-	+	++	--	---	---	---	+++

For the classification endpoints, ADMETLab 2.0 transforms the prediction probability values into six symbols: 0–0.1 (---), 0.1–0.3 (--), 0.3–0.5 (-), 0.5–0.7 (+), 0.7–0.9 (++), and 0.9–1.0 (+++).

## Data Availability

Data is contained within the article and Supplementary Materials.

## Supplementary Materials

The following supporting information can be downloaded at: https://www.mdpi.com/article/10.3390/ijms252312744/s1.
