# Supplementary material for: Enhancing Antifungal Drug Discovery Through Co-Culture with Antarctic Streptomyces albidoflavus Strain CBMAI 1855
Source: Int J Mol Sci. 2024 Nov 27;25(23):12744. doi: 10.3390/ijms252312744 (PMC11641416; doi:10.3390/ijms252312744)
Supplement: Supplementary file 1 [file ijms-25-12744-s001.zip › ijms-3198832-supplementary.pdf]

Supplementary Materials

Enhancing antifungal drug discovery through co-culture with Antarctic *Streptomyces albidoflavus* strain CBMAI 1855

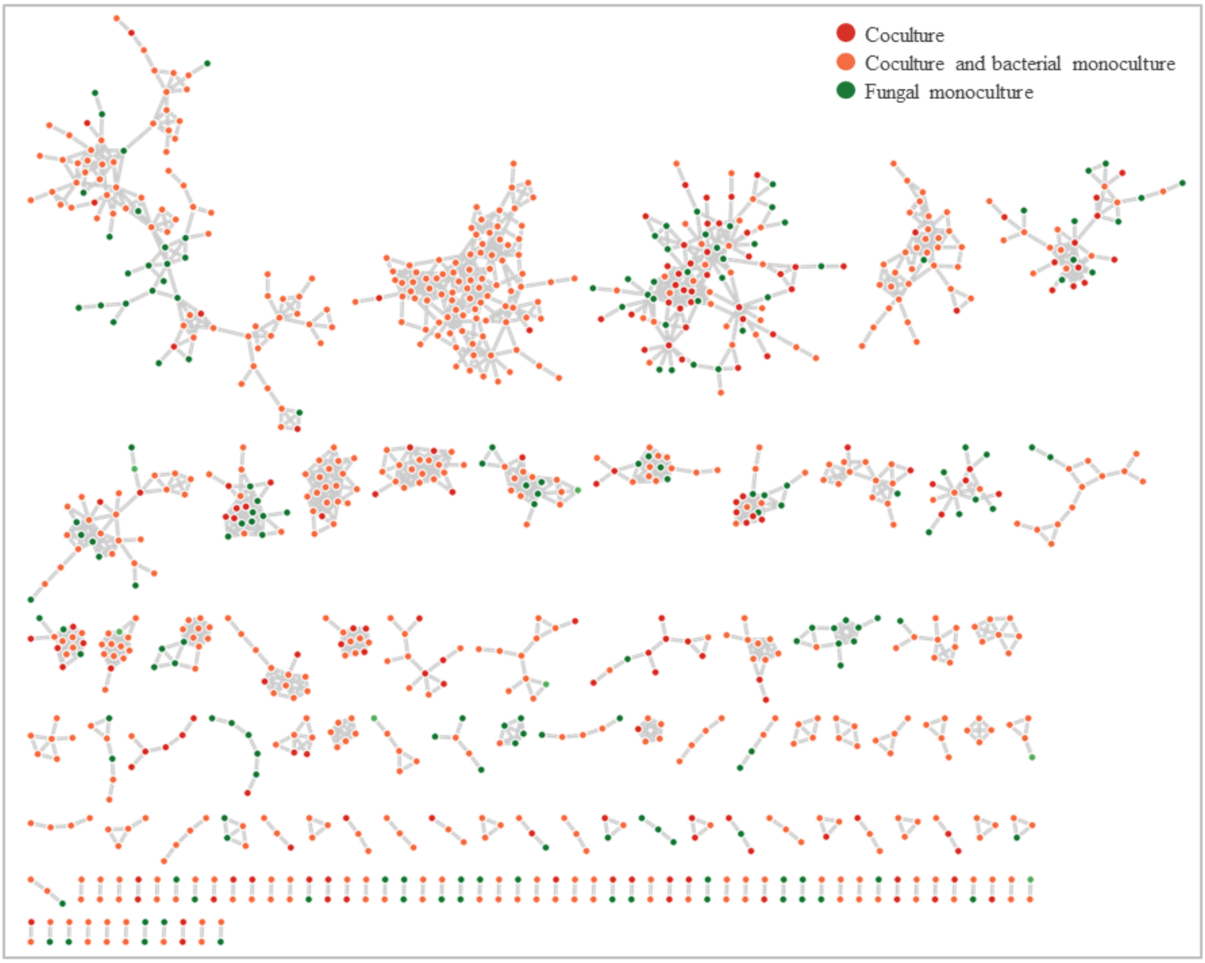

Figure S1: Molecular network illustrating the chemical space of co-culture, fungal monoculture, and bacterial monoculture treatments. The network was generated from the analysis of sample extracts with solvent blanks removed. Each node represents a consensus spectrum of a detected ion mass and is color-coded according to the treatment in which it was detected: co-culture (CBMAI 1855 and *A. flavus* ATCC 204304), fungal monoculture, and bacterial monoculture. For clarity, only nodes with at least two connections (clusters) are displayed.

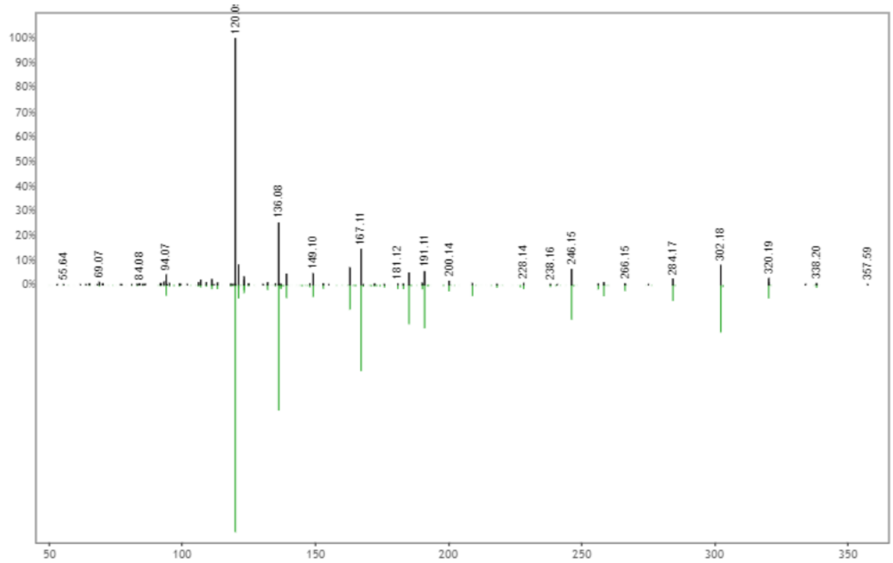

Figure S2. MS/MS match for 9-(4-aminophenyl)-3,7-dihydroxy-2,4,6-trimethyl-9-oxo-nonoic acid (candicidin related) between GNPS database (green) and CBMAI 1855 extracts (black).

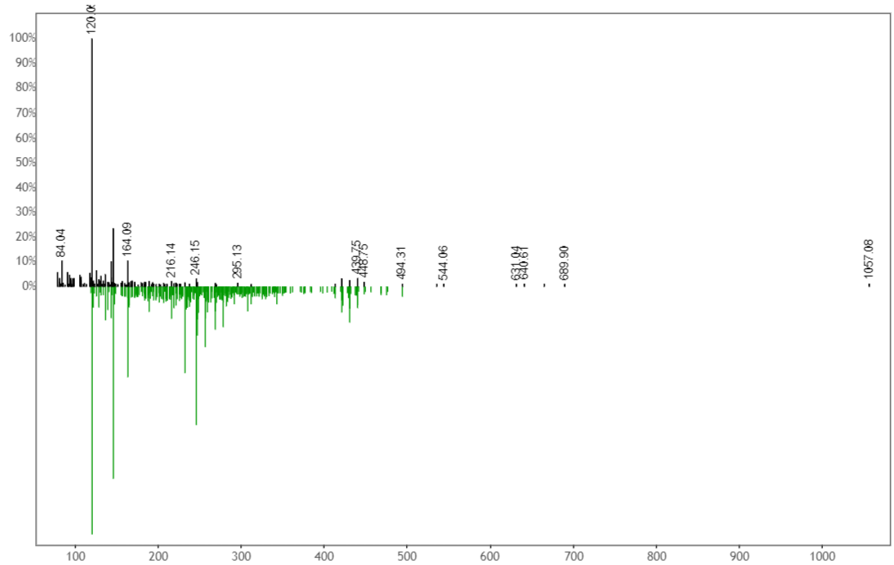

Figure S3. MS/MS match for Candicidin I between GNPS database (green) and CBMAI 1855 extracts (black).

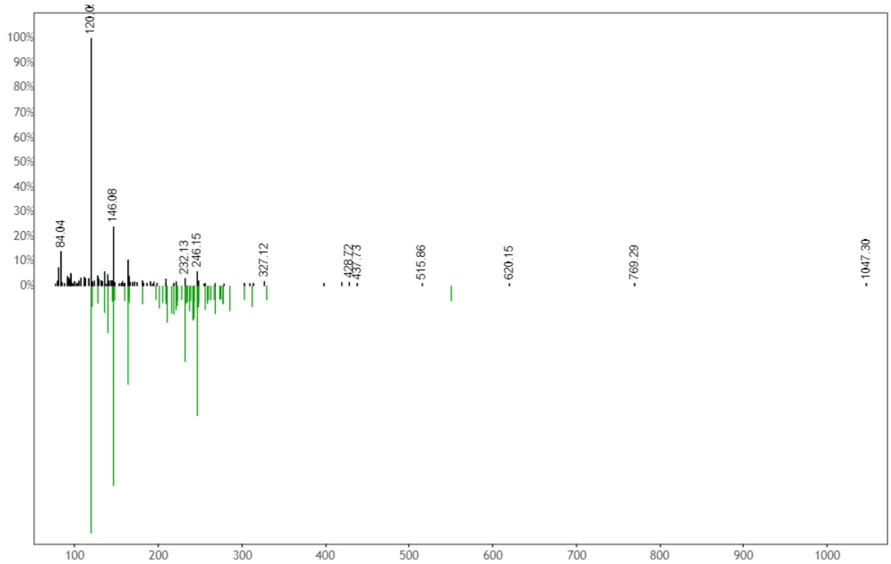

Figure S4. MS/MS match for Candicidin IV between GNPS database (green) and CBMAI 1855 extracts (black).

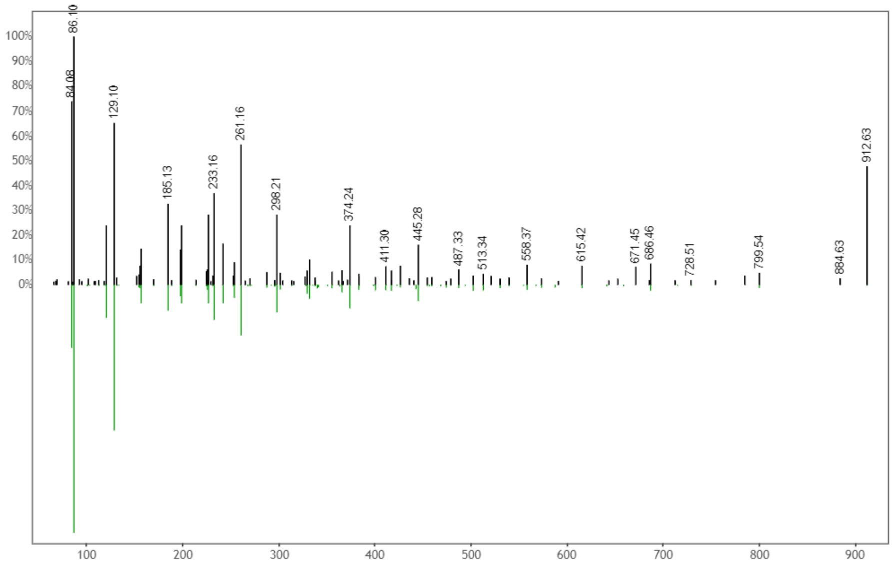

Figure S5. MS/MS match for Surugamide A between GNPS database (green) and CBMAI 1855 extracts (black).

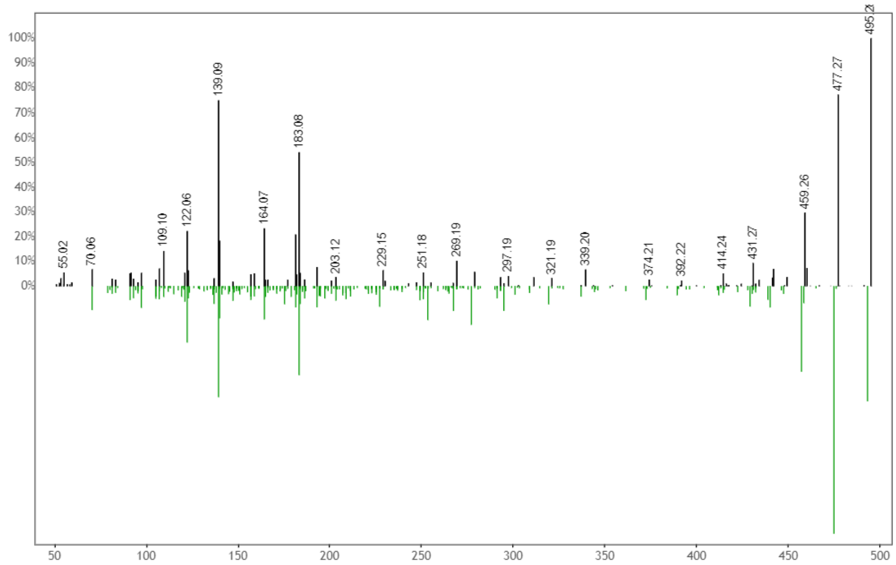

Figure S6. MS/MS match for Clifednamide A between GNPS database (green) and CBMAI 1855 extracts (black).

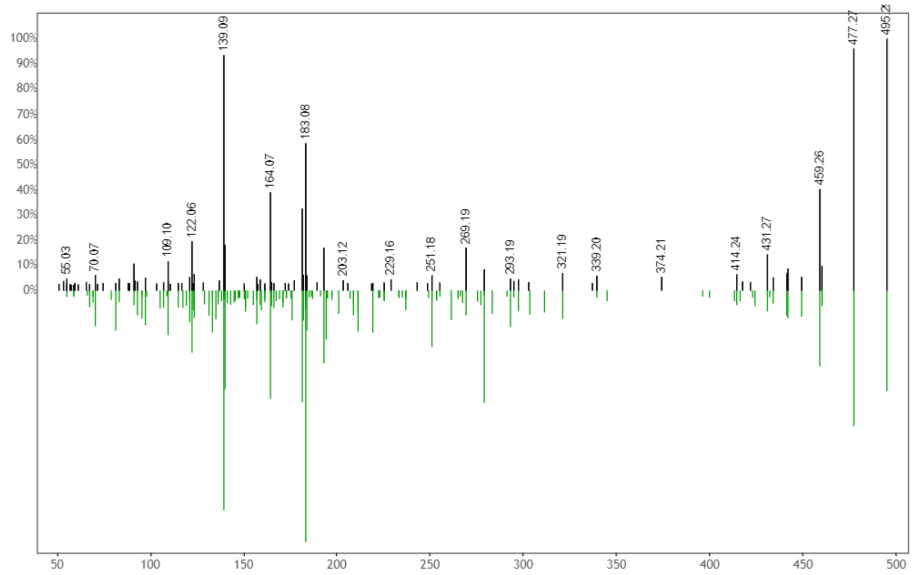

Figure S7. MS/MS match for Ikarugamycin epoxide between GNPS database (green) and CBMAI 1855 extracts (black).

Table S1. Comprehensive analysis of biosynthetic gene clusters (BGCs) in CBMAI 1855 identified by antiSMASH.

Region	Type	From	To	Most similar known cluster / Type		Similarity
**Region 1.1**	**NRPS**	**164,68**	**209,1**	**diisonitrile antibiotic SF2768**	**NRP**	**66%**
Region 1.2	terpene, NRPS-like, NRPS	229,45	305,1	valinomycin/montanastatin	NRP+Saccharide: Hybrid/tailoring saccharide	13%
**Region 1.3**	**T1PKS,NRPS**	**423,35**	**472,76**	**SGR PTMs**	**NRP+Polyketide**	**100%**
Region 1.4	terpene	503,12	529,68	hopene	Terpene	76%
Region 1.5	RiPP-like	608,81	619,02	hexacosalactone A	Other	4%
Region 2.1	RiPP-like	6,276	17,604			
Region 3.1	NI-siderophore	86,64	119,65	kinamycin	Polyketide	13%
Region 8.1	terpene	6,429	28,603	geosmin	Terpene	100%
Region 9.1	terpene	386,57	407,54	albaflavenone	Terpene	100%
Region 11.1	LAP, thiopeptide, RRE-containing	123,28	157,96	fluostatins M-Q	Polyketide	4%
Region 23.1	NRPS	85,449	135,74	WS9326	NRP	7%
Region 23.2	NRPS	173,8	236,04	dechlorocuracomycin	NRP	12%
**Region 24.1**	**NRPS**	**5,317**	**35,206**	**surugamide A/surugamide D**	**NRP**	**42%**
**Region 25.1**	**NRPS**	**1**	**41,377**	**surugamide A/surugamide D**	**NRP**	**19%**
**Region 27.1**	**NRPS, LAP**	**1**	**34,45**	**surugamide A/surugamide D**	**NRP**	**61%**
Region 30.1	NRPS	2,653	47,002	dudomycin A	NRP	17%

Region 31.1	NI-siderophore	3,775	33,595	desferrioxamin B	Other	100%
Region 34.1	ectoine	106,5	116,9	ectoine	Other	100%
Region 38.1	terpene	165,16	190,96	isorenieratene	Terpene	75%
Region 39.1	RiPP-like	18,856	25,3	streptamidine	RiPP: Other	41%
**Region 40.1**	**T3PKS**	**1,86**	**42,957**	**naringenin**	**Polyketide:Type III polyketide**	**100%**
**Region 40.2**	**T1PKS**	**47,893**	**101,94**	**candicidin**	**NRP+Polyketide**	**33%**
**Region 41.1**	**T1PKS**	**1**	**31,408**	**rosamicin**	**Polyketide**	**26%**
Region 42.1	T1PKS	1	5,181			
Region 43.1	T1PKS	1	4,749			
**Region 44.1**	**T1PKS,NRPS-like**	**1**	**59,019**	**candicidin**	**NRP+Polyketide**	**90%**
**Region 45.1**	**NRPS,T1PKS,lanthipeptide-class-ii,NRPS-like**	**1**	**74,671**	**antimycin**	**NRP:Cyclic depsipeptide+Polyketide:Modular type I polyketide**	**80%**
Region 46.1	transAT-PKS	1	7,989			
Region 47.1	T1PKS	1	22,674	rustmicin	Polyketide:Iterative type I polyketide	6%
Region 48.1	lassopeptide	74,379	96,762			

The table lists all detected BGCs, including their type, genomic coordinates, and the most similar known cluster with the corresponding biosynthetic product type and similarity percentage. Regions of interest discussed in the main text are highlighted, showing the diversity and potential of CBMAI 1855 to produce various natural products, including NRPs, polyketides, and terpenes. The BGC types are color-coded as follows: green for NRPS (Non-Ribosomal Peptide Synthetase), light blue for hybrids, red for NI-siderophore, purple for terpenes, navy blue for RiPP-like (Ribosomally synthesized and Post-translationally modified Peptides), and salmon for T1PKS (Type I Polyketide Synthase).
